# The Sufferings of Christ

**Published:** 1888-01-21

**Authors:** 


### WORDS OF CONSOLATION.

[Communications for this column should be addressed "Chaplain," care of the Editor of THE HOSPITAL, who will gratefully welcome any suggestions or inquiries from matrons, nurses, and the staff generally.]

### LXIII.—THE SUFFERINGS OF CHRIST.

*For Reading to the Sick.*

THE bodily sufferings of our blessed Lord increase every hour throughout the Passion. We pass on to the Cross itself, and what anguish do we not behold again in His sacred body. There could be no punishment like crucifixion for involving all the members in pain. We behold the hands pierced with cruel nails for the sins our hands must have answered for. In nearly every sin committed against the letter of the Sixth, Seventh, Eighth, and Ninth Commandments the hands have something to do. We have not only to take into account here positive acts of unkindness, impurity, dishonesty, but we may add perhaps some of us even things we have written which have pained them. Many sins are committed on paper in this way. Coldblooded and heartless insinuations are often conveyed by letter, involving the writers in deeper guilt than if they had sinned hastily with the tongue. Anonymous letters, falsified accounts, exaggerated advertisements, in all these matters the hands become the instruments of unrighteousness. Behold His feet also pierced and fixed for the sins which our feet have hurried us on to commit. Have we walked in the counsel of the ungodly, or stood in the way of sinners, then the feet have lent themselves for evil purposes. Consider next how He suffered through the eyes. Were they not blindfolded at one time, and for the rest constrained to gaze upon that scene of unrighteous bloodshed, every way they turned meeting with something to pain them. The eye was the first member of the body which yielded itself to unrighteousness when sin came into the world. "The woman saw that the tree was good for food and that it was pleasant to the eyes" (Gen. iii. 6.) Through lust of the eye sin very often begins to shape itself into actions after it has been conceived in the heart. We have eyes given us to see with; and those of us who are blest with good eyesight seldom think of the vast amount of pleasure derived through the use of these wonderfully delicate organs. May God forgive us the misuse of them. May He pardon the occasions when they have been knowingly turned when to have kept them straight would have also kept them pure. Yet again: For our sins contracted through listening to what we ought not to; for the conversations we have gladly consented to when we ought to have been ashamed to lend an ear at all; for the pleasure we have taken in hearing evil reports of others; yea, for our abuse of the sense of hearing our Lord had to endure the blasphemy of the multitude. He had to endure the sounds of wicked words, as they spoke all manner of evil falsely against Him, while it seemed as if His own prayers could not be heard. You remember what he says of three of those members we have now mentioned: the hand, the foot, the eye. "If one or other of these offend thee," that is, if it become the cause of sin, "cut it off and cast it from thee."

*(To be continued.)*

